# Do Chinese Children With Math Difficulties Have a Deficit in Executive Functioning?

**DOI:** 10.3389/fpsyg.2018.00906

**Published:** 2018-06-06

**Authors:** Xiaochen Wang, George K. Georgiou, Qing Li, Athanasios Tavouktsoglou

**Affiliations:** ^1^School of Business Administration, Zhejiang Gongshang University, Hangzhou, China; ^2^Department of Educational Psychology, University of Alberta, Edmonton, AB, Canada; ^3^Mental Health Center, Department of Social Sciences and Humanities, Zhejiang University of Media and Communication, Hangzhou, China; ^4^Faculty of Science, Concordia University of Edmonton, Edmonton, AB, Canada

**Keywords:** executive functioning, math disabilities, working memory, speed of processing, Chinese

## Abstract

Several studies have shown that Executive Functioning (EF) is a unique predictor of mathematics performance. However, whether or not children with mathematics difficulties (MD) experience deficits in EF remains unclear. Thus, the purpose of this study was to examine if Chinese children with MD experience deficits in EF. We assessed 23 children with MD (9 girls, mean age = 10.40 years), 30 children with reading difficulties and MD (RDMD; 12 girls, mean age = 10.82 years), and 31 typically-developing (TD) peers (16 girls, mean age = 10.41 years) on measures of inhibition (Color-Word Stroop, Inhibition), shifting of attention (Planned Connections, Rapid Alternating Stimuli), working memory (Digit Span Backwards, Listening Span), processing speed (Visual Matching, Planned Search), reading (Character Recognition, Sentence Verification), and mathematics (Addition and Subtraction Fluency, Math Standard Achievement Test). The results of MANOVA analyses showed first that the performance of the MD children in all EF tasks was worse than their TD peers. Second, with the exception of the shifting tasks in which the MD children performed better than the RDMD children, the performance of the two groups was similar in all measures of working memory and inhibition. Finally, covarying for the effects of processing speed eliminated almost all differences between the TD and MD groups (the only exception was Listening Span) as well as the differences between the MD and RDMD groups in shifting of attention. Taken together, our findings suggest that although Chinese children with MD (with or without comorbid reading difficulties) experience significant deficits in all EF skills, most of their deficits can be accounted by lower-level deficits in processing speed.

## Introduction

Several studies have reported that approximately 20% of school-age children experience mathematics difficulties (MD; see Gross-Tsur et al., 1996; Landerl and Moll, 2010; Geary, 2011; Moll et al., 2014). To better understand the cognitive underpinnings of MD, researchers have further examined the role of several candidate cognitive processes such as general cognitive ability (e.g., Toffalini et al., 2017), working memory (e.g., Passolunghi and Siegel, 2001; Swanson and Kim, 2007), speed of processing (e.g., Koontz and Berch, 1996; Compton et al., 2012), and phonological processing (e.g., Wise et al., 2008; Mazzocco and Grimm, 2013). One of the skills that remain largely unexplored is that of executive functioning (EF). Thus, the purpose of this study was to examine if children with MD experience deficits in EF.

EF is an umbrella term used to represent processes that allow individuals to respond flexibly to our environment and engage in deliberate, goal-directed behavior (e.g., Chan et al., 2008; Diamond, 2013). The three most studied EF skills^1^, particularly in relation to mathematics, are inhibition (the ability to suppress distracting information), shifting of attention (the ability to switch between mental sets, representations, and tasks), and working memory (the ability to store information for a short period of time and manipulate or process it)^2^. To date, several studies with typically developing (TD) children have shown that these three EF skills predict (jointly or independently) mathematics performance across a wide range of ages (e.g., Espy et al., 2004; Blair and Razza, 2007; Clark et al., 2010; Lan et al., 2011; Monette et al., 2011; van der Ven et al., 2012; McClelland et al., 2014; Viterbori et al., 2015; Chung et al., 2016; Purpura et al., 2017; see also Friso-van den Bos et al., 2013; Yeniad et al., 2013, for evidence from meta-analyses). In addition, there is some evidence that poor EF correlates with mathematics learning disabilities (e.g., Toll et al., 2011; Willoughby et al., 2016; Morgan et al., 2017).

There are several reasons why inhibition, shifting of attention, and working memory may relate to mathematics (Swanson and Beebe-Frankenberger, 2004; Censabella and Noël, 2008; Bull and Lee, 2014). First, inhibition may help individuals suppress the retrieval and use of developmental immature strategies, inappropriate number bonds (e.g., answering “18” to 3 + 6 = ?), or the use of information from a word problem that is irrelevant to the solution. Inhibition may also help working memory because inhibition of irrelevant information prevents working memory from becoming overloaded from this information. In turn, shifting of attention may help individuals alternate successfully between mathematical operations, solution strategies, and notations (e.g., between verbal digits, Arabic numerals, and non-symbolic quantity representations), or between the steps involved in solving a multistep problem. Finally, working memory may provide support for strategies such as verbal counting, the direct retrieval of arithmetic facts, the coordination of multiple steps in complex mathematics problems, and the maintenance of interim calculations during mental arithmetic.

Despite the volume of research examining the role of EF skills in TD children (see e.g., Bull and Lee, 2014; Cragg and Gilmore, 2014, for a review), far less is known about the role of EF skills in MD. In addition, the few studies that compared children with MD to TD children have produced mixed findings. Passolunghi et al. (1999; see also Passolunghi and Siegel, 2001, 2004) have found that children who were poor problem solvers were performing significantly lower than good problem solvers in working memory tasks. In addition, as a group, they were committing more intrusion errors (i.e., they recalled non-target information more often) in a Listening Span task. Based on these findings, Passolunghi and colleagues concluded that the working memory deficits exhibited by children with poor problem solving skills can be traced to more fundamental deficits in inhibition. Because children with MD could not inhibit irrelevant information, more information (relevant or not) was kept active in working memory, which, in turn, overloaded its capacity. Fuchs and colleagues (e.g., Fuchs et al., 2005, 2006) also reported that children with MD had elevated levels of inattentive behavior (based on teacher ratings of attentional skills) and that inattentive behavior, along with working memory deficits, predicted the emergence of computational and problem-solving mathematical difficulties over the course of Grade 1. In contrast to these findings, some studies have shown that children with MD do not experience deficits in inhibition (e.g., van der Sluis et al., 2004; Censabella and Noël, 2005; de Weerdt et al., 2013; McDonald and Berg, 2017). For example, van der Sluis et al. (2004) found that children with MD differed from the control group only on tasks involving both inhibition and shifting, and not on tasks involving only inhibition.

Similar contradictory findings have been reported for working memory, and each one of its components (central executive, phonological loop, and visuo-spatial sketchpad; see Passolunghi, 2006, for a review). For example, whereas some researchers have shown that children with MD experience deficits in central executive (e.g., Geary et al., 2000; Passolunghi and Siegel, 2001; Swanson and Sachse-Lee, 2001; Cai et al., 2013), others did not (e.g., McLean and Hitch, 1999; Schuchardt et al., 2008; McDonald and Berg, 2017). Likewise, whereas some researchers have reported significant deficits among children with MD in phonological loop (e.g., Geary et al., 1991; Swanson and Sachse-Lee, 2001; van der Sluis et al., 2005; Cai et al., 2013) and in visuo-spatial sketchpad (e.g., McLean and Hitch, 1999; D’Amico and Guarnera, 2005; Berg, 2008; Cai et al., 2013; Szűcs et al., 2013), others did not (e.g., Bull et al., 1999; Geary et al., 2000, 2004; Landerl et al., 2004).

Several issues need to be considered when interpreting these conflicting results. The first issue relates to speed of processing and whether its effects are partialled out or not before testing for differences between groups in EF. Some studies have shown that children with MD process information more slowly than TD children (e.g., Bull and Johnston, 1997; Swanson and Sachse-Lee, 2001; Chan and Ho, 2010; Vukovic and Siegel, 2010; however, see also Berg, 2008). Because speed of processing is a strong predictor of mathematics performance (e.g., Bull and Johnston, 1997; Fuchs et al., 2006; Peng et al., 2016; Cui et al., 2017), it is possible that MD persist because of persistent deficits in speed of processing, which hinder automatic fact retrieval from long-term memory.

Relatedly, little attention has been paid on how speed of processing has been operationalized in different studies. Some researchers have used naming speed tasks (i.e., digit naming) as measures of speed of processing (e.g., Geary et al., 2007; Chan and Ho, 2010; Vukovic and Siegel, 2010; Moll et al., 2016). Using a naming speed task as a measure of processing speed is problematic because of evidence showing that measures of naming speed do not load on the same factor as measures of speed of processing (e.g., Visual Matching, Cross-out; see van den Bos et al., 2003; Bowey et al., 2004). In addition, poor performance in a naming speed task does not necessarily mean that children experience difficulties in processing speed. Poor performance in a naming speed task may also reflect low-quality phonological representations (e.g., Elbro, 1998), problems in simultaneously processing multiple stimuli when they are presented in serial fashion (e.g., Protopapas et al., 2013), or even problems in forming a “perceptual anchor” in tasks that involve a small set of repeated stimuli (e.g., Ahissar, 2007). Second, because most of the EF tasks (particularly the inhibition and shifting of attention tasks) are speeded and because MD children are often selected based on their poor performance in calculation fluency tasks, this may give rise to EF difficulties. Thus, unless the effects of speed of processing are controlled, we cannot exclude the possibility that the observed difficulties of children with MD in EF tasks are due to lower-level processing speed deficits (see van der Sluis et al., 2004, for a similar argument). In the present study we used more conventional measures of processing speed and we also partialled out the effects of speed of processing prior to testing for group differences in EF skills.

The second issue relates to comorbidity between reading and mathematics. Math difficulties often co-occur with reading difficulties in children with learning disabilities (Gross-Tsur et al., 1996; Moll et al., 2014). Some researchers have argued that children with MD are cognitively different from children with RDMD (e.g., Geary et al., 2007; Landerl et al., 2009; Compton et al., 2012). Because most previous studies did not distinguish between children with MD and children with RDMD (e.g., Geary et al., 2007; Berg, 2008; Censabella and Noël, 2008; Cai et al., 2013), the discrepant findings may reflect a mixed pattern of EF deficits for children with MD and children with RDMD.

It is also worth noting that most previous studies on EF and MD have been conducted in North America or in Europe (see list of studies in the meta-analyses by Friso-van den Bos et al., 2013 and Yeniad et al., 2013), and we do not know if their findings generalize to an East Asian country (e.g., China). We have several reasons to believe that the findings may be different. First, some cross-cultural studies have shown that Chinese children perform better than North American children not only on mathematics skills (e.g., Zhao et al., 2014; Lonnemann et al., 2016; see also Wang and Lin, 2009), but also on EF skills (e.g., Sabbagh et al., 2006; Lan et al., 2011). However, the superior performance of Chinese children in both skills has not led to stronger effects of EF on mathematics skills. Lan et al. (2011), for example, found that whereas inhibition was a unique predictor of calculation ability among American preschoolers, it was not among Chinese preschoolers. Second, Geary et al. (2000) found that American children with MD (with or without comorbid reading difficulties) committed more counting string errors (e.g., recalling the number following one of the addends in the counting string) than their TD peers. They attributed this to inefficient inhibition of irrelevant associations. However, Chinese children practice simple additions and subtractions from the age of 3 (Cheng et al., 2001) and by the time they go to elementary school, they are expected to retrieve the answer to simple calculation problems from their long-term memory. Consequently, Chinese children with MD may not experience deficits in inhibition. In line with this hypothesis, Peng et al. (2012) found that performance in a color-word Stroop task (one of the most widely used measures of inhibition) did not differentiate Chinese fifth-graders with MD from their TD peers. Finally, the Chinese linguistic system (e.g., short pronunciation of numbers in Chinese and regular number naming structure; see Ng and Rao, 2010, for details) may increase the working memory capacity and reduce the working memory difficulties in Chinese children with MD. Given that only two studies to date have examined the role of EF skills in MD in China (Chan and Ho, 2010; Peng et al., 2012) and none of them has controlled for the effects of speed of processing, more research is needed on this topic.

## Materials and Methods

### Participants

The participants in this study were 84 Chinese children (37 girls, 47 boys; mean age = 10.56 years, *SD* = 1.11) attending five inner-city public schools in Hangzhou. Because there is no formal diagnosis and coding of children as math or reading disabled in China, to select our participants we used the following two-step approach: First, we administered a calculation fluency task (Addition and Subtraction Fluency from WIAT-II; Wechsler, 2009) and a reading fluency task (Sentence Verification; Lei et al., 2011) to a large group of Grade 4, 5, and 6 children (*n* = 1,160; 570 girls and 590 boys). Both tasks were administered to the whole classroom by our trained graduate students.

Second, based on the performance of the children in these two screening measures, we carefully selected three groups of children according to the following criteria: children in the control group had to score at or above the 35th percentile of their grade level in both arithmetic and reading fluency tasks. Children with MD only had to score below the 20th percentile of their grade level in arithmetic fluency (i.e., ≤raw score of 79 in Grade 4; ≤raw score of 92 in Grade 5; ≤raw score of 93 in Grade 6) and above the 35th percentile of their grade level in reading fluency (i.e., ≥raw score of 59 in Grade 4; ≥raw score of 66 in Grade 5; ≥raw score of 67 in Grade 6). Finally, children with both mathematics and reading difficulties had to score below the 20th percentile of their grade level in both mathematics and reading^3^.

From this selection procedure, we first identified 33 children with poor reading and poor mathematics performance and 25 children with poor mathematics and good reading performance. Three children from the former and two children from the latter group had a non-verbal IQ lower than 85 (assessed with Raven’s Matrices) and were excluded from further testing. Thus, our final sample consisted of 30 children with poor mathematics and poor reading performance (18 boys, 12 girls; 10 from Grade 4, 8 from Grade 5, and 12 from Grade 6) and 23 children with poor mathematics and good reading performance (14 boys, 9 girls; 9 from Grade 4, 7 from Grade 5, and 7 from Grade 6). Next, to select the children with no reading or MD, we randomly sampled one twentieth of the children meeting the selection criterion (a score higher than the 35th percentile in both reading and mathematics) in each grade^4^. This resulted in 32 children. A child with a non-verbal IQ score lower than 85 was further eliminated leaving us with a sample of 31 children (15 boys, 16 girls; 12 from Grade 4, 10 from Grade 5, and 9 from Grade 6). Parental permission and ethical approval from the Research Ethics Committee of the Zhejiang Gongshang University was obtained prior to testing. Descriptive statistics on age, non-verbal IQ, reading fluency, and arithmetic fluency tasks are presented in **Table 1**.

**Table 1:** Descriptive statistics on the screening measures separately for each group.

	TD (*n* = 31)	MD (*n* = 23)	RDMD (*n* = 30)		
			
	*M* (*SD*)	*M* (*SD*)	*M* (*SD*)	*F*	Group comparisons
Age (years)	10.50 (0.86)	10.40 (1.24)	10.82 (1.11)	1.05	TD = RDMD = MD
Raven	105.42 (6.35)	102.30 (5.57)	102.38 (5.34)	1.41	TD = RDMD = MD
Sentence Verification	72.29 (4.58)	69.35 (6.54)	38.60 (6.92)	227.44^∗∗∗^	TD = MD > RDMD
Character Recognition	142.65 (3.77)	139.43 (8.48)	124.50 (9.06)	51.34^∗∗∗^	TD = MD > RDMD
Calculation Fluency	135.45 (17.32)	80.00 (10.02)	70.93 (17.11)	115.50^∗∗∗^	TD > MD = RDMD
MSAT	35.61 (7.49)	32.70 (7.25)	29.77 (5.86)	5.51^∗∗∗^	TD > MD = RDMD

*TD, typically developing group; MD, mathematics difficulties group; RDMD, reading and mathematics difficulties group. *^∗∗∗^p* < 0.001*.

### Materials

#### Non-verbal IQ

Non-verbal IQ was assessed with Raven’s Standard Progressive Matrices (Raven et al., 2016). Children were presented with a pattern of shapes/geometric designs that was missing a piece and were asked to choose among six to eight alternatives the piece that would accurately complete the pattern. The task consisted of five sets of 12 items (total of 60). A child’s score was the total number correct. Cronbach’s alpha reliability coefficient in our sample was 0.89.

#### Speed of Processing

To assess speed of processing we administered two measures: Visual Matching and Planned Search. In Visual Matching, children were required to find and underline the two numbers that were the same in each of the eight rows in a card. There were six numbers of the same length in each row (e.g., 6, 2, 9, 6, 7, 1). In Card 1, the first four rows contained 2-digit numbers and the last four 3-digit numbers. In Card 2, the first four rows contained 4-digit numbers and the last four 5-digit numbers. Maximum time allowed per card was 180 s. A participant’s score in Cards 1 and 2 was the total time divided by the number of correct responses. Cronbach’s alpha reliability coefficient in our sample was 0.85. In Planned Search, children were asked to match as quickly as possible a target object, number, or letter (located inside a box in the middle of a visual field) with the same object, number, or letter that was located in the visual field among distractors. Each item consisted of two searches, one on the top half of the page and one on the bottom half of the page. Each target had only one match on a page. We recorded the time to complete each search on each page and the participant’s score was the total time to complete all searches. Cronbach’s alpha reliability coefficient in our sample was 0.79.

#### Executive Functioning

##### Inhibition

Inhibition was assessed with the Expressive Attention task from DN CAS-2 (Naglieri et al., 2014) and the Inhibition task from NEPSY-II (Korkman et al., 2007). In Expressive Attention, children were presented with three pages of stimuli. In the first page, children were asked to read color words [i.e., 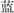 (blue), 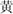 (yellow), 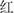 (red), 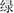 (green)] that were semi-randomly arranged in eight rows of five. In the second page, children were asked to name an array of color patches of the aforementioned colors. In the third page, children were asked to name as fast as and as accurately as possible the color of the ink in which color words were printed [e.g., the word 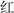 (Red) printed in blue ink] instead of saying the color word. Before each timed trial, the children were presented with a practice page to ensure they understood the instructions. A participant’s score was the time to finish the third page. Cronbach’s alpha reliability coefficient in our sample was 0.77. In the Inhibition task, children were required to look at a series of black and white shapes or arrows and name the shape (e.g., say square or circle), the direction (e.g., say up or down), or the opposite (e.g., when you see a square shape, say circle; and when you see a circle shape, say square), depending on the color of the shape or arrow. The completion time in seconds for the test items in each condition (i.e., Naming, Inhibition, and Switching) was recorded. A participant’s score was the total time to finish the Inhibition task. Cronbach’s alpha reliability coefficient in our sample was 0.82.

##### Shifting

Shifting was assessed with the Planned Connections task from the DN CAS-2 battery (Naglieri et al., 2014) and the Rapid Alternating Stimuli (RAS) task from the RAN/RAS test battery (Wolf and Denckla, 2005). Planned Connections is a transparent adaptation of Trail Making (Reitan and Wolfson, 1992). In this task, children were presented with four pages of numbers (1–13) and letters (A–M), and, in each page, they were asked to connect the numbers to the letters in successive order (1, A, 2, B, 3, C, etc.) as fast and as accurately as possible. The score was the total time to finish all pages. Cronbach’s alpha reliability in our study was 0.80. In RAS, children were required to name as fast and as accurately as possible color patches mixed up with digits (i.e., blue, 2, yellow, 6, green, 9, black, 7, etc.). The color patches and digits were randomly presented in five rows of ten. Prior to testing, the children were asked to name each of the RAS stimuli in a practice trial to ensure familiarity. A participant’s score was the total time to name all items. Wolf and Denckla (2005) reported test-retest reliability for this task to be 0.84.

##### Working memory

The Digit Span Backwards task and the Listening Span task were used to assess working memory. The Digit Span Backwards task was adopted from WISC-III (Wechsler, 1992). Children were asked to repeat a sequence of digits in the reverse order. The strings of digits were presented orally by the experimenter with a time interval of about 1 second between each digit. The strings started with only two digits and one digit was added at each difficulty level (the maximum length was seven digits). The task was terminated when children failed both trials of a given length. The children’s score was the number of digit strings accurately recalled. Cronbach’s alpha reliability coefficient in our sample was 0.75. The Listening Span task was adapted in Chinese from Daneman and Carpenter (1980). The children listened to groups of sentences and were asked to determine if each sentence was true or false (e.g., “All mothers work in an office”). Children were instructed to keep the last word in each sentence in their memory and then after completing a sentence group they were asked to say the last word in each sentence in the same order. A participant’s score was the total number of sets correctly recalled (max = 5). Cronbach’s alpha reliability coefficient in our sample was 0.81.

#### Arithmetic Skills

##### Arithmetic accuracy

The Math Standard Achievement Test (MSAT) from Dong and Lin (2011) was used to assess arithmetic accuracy. The task has been used in previous studies in China showing good psychometric properties (e.g., Cai et al., 2013, 2018). The test included 30 items: 26 items were multiple choice questions (e.g., If 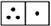 is number 31, what number is 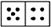 ? 4, 9, 45, or 54?) and 4 items were fill-in questions (e.g., Based on the map you have in front of you, how long will it take Fang to go to the bookstore, if he first passes by Li’s home?). The task was discontinued after four consecutive errors. A participant’s score was the total number correct. Cronbach’s alpha reliability coefficient in our sample was 0.84.

##### Arithmetic fluency

To assess arithmetic fluency we administered the addition and subtraction fluency tasks from WIAT-III (Wechsler, 2009). In each subtest, children were asked to solve as many additions or subtractions as possible within a 1-min time limit by writing their response in the space provided beside each problem. Each subtest included two pages (24 items on each page; total of 48 problems). A participant’s score was the total correct number of additions and subtractions completed within the time limit. The scores in addition fluency correlated 0.85 with the scores in subtraction fluency.

#### Reading Skills

##### Reading accuracy

Character Recognition was adopted from Li et al. (2012) to assess reading accuracy. The task has been used in previous studies in Chinese showing good reliability and validity evidence (e.g., Xue et al., 2013; Zhang et al., 2013; Liao et al., 2015). Children were asked to read aloud 150 Chinese two-character words that were arranged in terms of increasing difficulty. The task was discontinued after 15 consecutive errors. A participant’s score was the total number correct. Cronbach’s alpha reliability coefficient in our sample was 0.89.

##### Reading fluency

Sentence Verification from Lei et al. (2011) was used to assess reading fluency. The task has been used in previous studies in Chinese showing good psychometric properties (e.g., Liao et al., 2014; Pan et al., 2016; Xia et al., 2018). Children were asked to read silently simple sentences and indicate if the meaning of each sentence was true or false by circling Y (for Yes) or N (for No) printed at the end of each sentence (e.g., *Horse is an animal*. Y – N). The semantic content and linguistic format of each sentence was simple so that only very basic comprehension processes were required. A 3-min time limit was implemented. A participant’s score was the total number of correctly answered sentences.

### Procedure

Testing was conducted by the first and third authors, and six graduate students who received extensive training on test administration and scoring. Sentence Verification, and Addition and Subtraction Fluency were administered in a group setting. The rest of the tasks were administered to each child individually during school hours in a quiet room at school. Individual testing took place 3 weeks following the group testing and lasted approximately an hour.

## Results

### Group Comparisons on Inhibition

First, we ran a MANOVA with the two inhibition tasks as dependent variables and group as a fixed factor. The results revealed a main effect of group, Wilk’s λ = 0.610, *F*(4,160) = 11.23, *p* < 0.001. Follow-up ANOVAs showed that the groups differed in both Expressive Attention [*F*(2,81) = 18.85, *p* < 0.001] and Inhibition [*F*(2,81) = 18.90, *p* < 0.001]. *Post hoc* analyses showed that the TD group performed better than the MD and RDMD groups in both Expressive Attention and Inhibition (see **Table 2**). No significant differences were found between the MD and the RDMD groups.

**Table 2:** Descriptive statistics on the EF tasks and group comparisons.

	TD (*n* = 31)		MD (*n* = 23)		RDMD (*n* = 30)					Processing speed as a covariate		
	*M*	*SD*	*M*	*SD*	*M*	*SD*	*F*(2,81)	η*^2^*	*Post hoc*	*F*(2,81)	η^2^	*Post hoc*
**Inhibition**												
Exp. Attention	49.88	9.44	63.56	15.83	72.13	16.91	18.85^∗∗∗^	0.32	TD < MD = RDMD	4.62^∗^	0.10	TD = MD < RDMD
Inhibition	30.16	4.15	36.84	6.93	41.27	9.31	18.90^∗∗∗^	0.32	TD < MD = RDMD	3.99^∗^	0.09	TD = MD < RDMD
**Shifting**												
Pl. Connections	37.57	6.36	51.02	18.24	60.36	14.89	21.65^∗∗∗^	0.35	TD < MD < RDMD	4.83^∗^	0.11	TD = MD, RDMD = MD, TD < RDMD
RAS	29.90	4.87	37.62	9.86	44.45	12.29	17.80^∗∗∗^	0.31	TD < MD < RDMD	4.84^∗^	0.11	TD = MD, RDMD = MD, TD < RDMD
**Working memory**												
DSB	8.16	2.56	6.39	1.95	5.67	1.92	10.46^∗∗∗^	0.21	TD > MD = RDMD	3.12^∗^	0.07	TD = MD, RDMD = MD, TD > RDMD
Listening Span	3.03	0.84	2.43	0.90	2.23	1.16	8.04^∗∗^	0.17	TD > MD = RDMD	5.60^∗^	0.15	TD > MD = RDMD
**Processing speed**												
Visual Matching	48.58	4.50	42.83	5.254	39.83	5.849	22.03^∗∗∗^	0.35	TD > MD = RDMD			
Planned Search	7.20	1.31	8.49	2.65	8.60	1.70	4.99^∗∗^	0.11	TD < MD = RDMD			

*TD, typically developing group; MD, mathematics difficulties group; RDMD, reading and mathematics difficulties group; DBS, Digit Span Backwards; RAS, Rapid Alternating Stimuli. ^∗^*p* < 0.05; ^∗∗^*p* < 0.01; ^∗∗∗^*p* < 0.001*.

### Group Comparisons on Shifting

A MANOVA with the two shifting tasks as dependent variables and group as a fixed factor revealed a main effect of group, Wilk’s λ = 0.579, *F*(4,160) = 12.57, *p* < 0.001. Follow-up ANOVAs showed that the groups differed in both Planned Connections [*F*(2,81) = 21.65, *p* < 0.001] and RAS [*F*(2,81) = 17.80, *p* < 0.001]. *Post hoc* analyses showed that the TD group performed better than the MD and RDMD groups in both Planned Connections and RAS. The MD group also performed significantly better than the RDMD group (see **Table 2**).

### Group Comparisons on Working Memory

Next, we ran a MANOVA with the two working memory tasks as dependent variables and group as a fixed factor. The results revealed a main effect of group, Wilk’s λ = 0.745, *F*(4,160) = 6.34, *p* < 0.01. Follow-up ANOVAs showed that the groups differed in both Digit Span Backwards [*F*(2,81) = 10.46, *p* < 0.001] and Listening Span [*F*(2,81) = 8.04, *p* < 0.01]. *Post hoc* analyses showed that the TD group obtained significantly higher scores than the MD and RDMD groups in both Digit Span Backwards and Listening Span. There was no significant difference between the MD and RDMD groups (see **Table 2**).

### Group Comparisons on Processing Speed

Finally, we ran a MANOVA with the two processing speed tasks as dependent variables and group as a fixed factor. The results revealed a main effect of group, Wilk’s λ = 0.640, *F*(4,160) = 10.01, *p* < 0.001. Follow-up ANOVAs showed that the groups differed in both Visual Matching [*F*(2,81) = 22.03, *p* < 0.001] and Planned Search [*F*(2,81) = 4.99, *p* < 0.05]. *Post hoc* analyses showed that the TD group obtained significantly higher scores than the MD and RDMD groups in both Visual Matching and Planned Search. Again, there was no significant difference between the MD and RDMD groups (see **Table 2**).

### Group Comparisons on Executive Functioning After Controlling for Processing Speed

Finally, we performed three MANCOVAs (one for each EF skill) covarying for the effects of processing speed (Visual Matching and Planned Search) (see **Table 2**). In terms of inhibition, the results revealed a main effect of group, Wilk’s λ = 0.861, *F*(4,158) = 3.07, *p* < 0.001. Follow-up ANCOVAs showed that the groups differed in both Expressive Attention [*F*(2,80) = 4.62, *p* < 0.05] and Inhibition [*F*(2,80) = 3.99, *p* < 0.05]. *Post hoc* analyses revealed no significant differences between the TD and MD groups. In addition, both groups performed significantly better than the RDMD group.

In terms of shifting, the results of MANCOVA revealed a main effect of group, Wilk’s λ = 0.832, *F*(4,158) = 3.80, *p* < 0.001. Follow-up ANCOVAs showed that the groups differed in both Planned Connections [*F*(2,80) = 4.83, *p* < 0.05] and RAS [*F*(2,80) = 4.84, *p* < 0.05]. *Post hoc* analyses revealed that the only significant difference was between the TD and the RDMD groups. The MD group did not differ significantly from the TD and RDMD groups.

Finally, in terms of working memory, the results of MANCOVA revealed a main effect of group, Wilk’s λ = 0.858, [*F*(4,158) = 3.14, *p* < 0.001]. Follow-up ANCOVAs showed that the groups differed in both Digit Span Backwards [*F* (2,80) = 3.12, *p* < 0.05] and Listening Span, [*F*(2,80) = 5.60, *p* < 0.05]. *Post hoc* analyses showed no significant differences between the TD and MD groups in Digit Span Backwards, but significant difference between the two groups in Listening Span. The MD group did not differ from the RDMD group on either working memory task.

## Discussion

Several studies have demonstrated that EF is an important predictor of mathematics performance and a risk factor of MD (see Bull and Lee, 2014; Cragg and Gilmore, 2014, for reviews). Nevertheless, because EF consists of several sub-components (inhibition, shifting, and working memory; Miyake et al., 2000) and because MD overlaps with reading difficulties (Landerl and Moll, 2010), it remains unclear if MD children have a deficit in all EF sub-components and if these deficits are accentuated by concomitant difficulties in reading disabilities. Thus, in this study, we sought to examine the nature of EF deficits in Chinese children with mathematics disabilities (with or without comorbid reading difficulties).

First, our results showed that the MD children differed from their TD peers on all EF skills (see Chan and Ho, 2010; Cai et al., 2013, for similar findings). However, most differences disappeared once we controlled for the effects of speed of processing. Notably, the only difference between the TD and MD groups that remained significant was in Listening Span. This suggests that the significant differences between MD and TD children in inhibition or shifting of attention that have been reported in previous studies (e.g., Geary et al., 2000, 2007; Szűcs et al., 2013; McDonald and Berg, 2017) may reflect differences between groups in speed of processing more so than in EF. The argument put forward by some researchers that deficits in inhibition are likely responsible for the observed deficits of MD children in working memory (e.g., Passolunghi et al., 1999; Passolunghi and Siegel, 2001) does not seem to be supported by our findings either, because the MD group continued to perform more poorly than the TD group in Listening Span despite their equal performance in inhibition (that is after controlling for processing speed).

The absence of a significant difference between the TD and MD groups in our study may also reflect cultural differences. More specifically, because Chinese children go to school at the age of 3 and they systematically practice simple additions/subtractions, by the time they go to Grade 1 they are able to retrieve the answer to simple calculation problems from their memory (Geary et al., 1996). This likely reduces interference from competing responses and decreases the effect of inhibition in mathematics. In addition, because Chinese digits have a shorter pronunciation duration than digits in other languages such as English (digits in China are monosyllabic) and because shorter names allow for a greater number of digits to be stored in working memory, this may explain why we did not observe any differences between the MD and TD groups in Digit Span Backwards.

Second, our findings replicate those of previous studies in North America/Europe (e.g., Swanson, 1993; Geary et al., 2000; Passolunghi and Siegel, 2001; Swanson and Sachse-Lee, 2001; Andersson and Lyxell, 2007) showing persistent deficits of the MD group in working memory (at least on measures of the central executive such as Listening Span). Although similar differences were detected in Digit Span Backwards, they did not survive the statistical control of speed of processing. An explanation may relate to the nature of the Digit Span Backwards task. More specifically, some researchers have argued that although it is frequently used as a measure of working memory, it is relatively shallow in its processing demands (e.g., St Clair-Thompson, 2010; Georgiou and Das, 2016).

Some limitations of the present study are worth mentioning. First, our participants did not come with a formal diagnosis of learning disabilities. Such a diagnosis does not exist in China. That is also why we selected our participants by first screening a relatively large sample of children. Second, we screened our participants using reading and mathematics fluency tasks in a group setting. Despite the fact that this is a convenient approach and it has been used in several previous studies to screen children for learning disabilities, we acknowledge that it comes with limitations (e.g., some children may get distracted in the presence of other students and may not invest the maximum of their effort). Nonetheless, we obtained similar differences between groups in the two individually administered measures (Character Recognition and MSAT), which allows us to say with some confidence that our selection worked quite well. Third, we did not manipulate the modality of the EF tasks (i.e., verbal vs. quantity) in our study. For example, some previous studies have used either verbal or numerical EF tasks to rule out the possibility that EF deficits manifest themselves only within a specific domain (e.g., Peng et al., 2013). Fourth, because of limited resources, we did not assess children with only reading difficulties. We acknowledge that this would have strengthened the findings of our study. Finally, we espoused a rather narrow view of EF consisting of three core components. That is why we did not administer measures of planning or visual-spatial memory and we did not assess other types of inhibition that are often operationalized with Go/No-go tasks. Future studies may assess EF more broadly.

## Conclusion

To conclude, our findings add to a growing body of research on the role of EF skills in MD (e.g., Swanson, 1993; Passolunghi and Siegel, 2001; van der Sluis et al., 2004; Geary et al., 2007; Compton et al., 2012; Peng et al., 2012) highlighting the role of speed of processing as a mediating factor in the severity of EF difficulties. Importantly, although our MD group differed from the TD group on all EF tasks, after controlling for speed of processing, the only difference that remained significant was in Listening Span. Although we are not the first ones to report non-significant differences between children with and without MD in inhibition and/or shifting of attention (see e.g., van der Sluis et al., 2004; Censabella and Noël, 2008 for similar findings), our study is the first one to show this in mainland China. We argue here that the linguistic features of Chinese (i.e., short pronunciation of digits in Chinese, transparent number-naming system), the age at which children start learning to do simple calculations, and the increased levels of parental involvement in children’s learning (particularly in mathematics; see Deng et al., 2015) may alleviate the negative impact of EF difficulties in MD in China. Future studies may examine the role of different EF skills in MD across cultures.

## Author Contributions

XW, GG, and AT designed the study. XW and QL helped in the data collection and data analysis, and also wrote the “Materials and Methods” and “Results” section of the paper. GG wrote the “Introduction” and “Discussion” sections of the paper. AT helped in revising the whole manuscript.

## Conflict of Interest Statement

The authors declare that the research was conducted in the absence of any commercial or financial relationships that could be construed as a potential conflict of interest.

## Footnotes

1 We acknowledge that some researchers use the term in a broader way and include under the umbrella of EF skills such cognitive flexibility, verbal fluency, and planning (e.g., Latzman and Markon, 2010; Testa et al., 2012).

2 Notice that inhibition, shifting of attention, and working memory are also broad terms (Nigg, 2000; Friedman and Miyake, 2004; Wager et al., 2006; Baddeley, 2012). For example, working memory consists of four components: central executive, phonological look, visuo-spatial sketchpad, and episodic buffer (Baddeley, 2012).

3 The 20th and 35th percentiles are commonly used as cutoff scores to select participants with and without reading/mathematics difficulties (e.g., Badian, 1999; Landerl et al., 2004; Fuchs et al., 2008; Tang, 2012). However, as indicated in Swanson and Jerman’s (2006) meta-analysis, measures used to establish math disabilities vary from the 48th percentile to the 8th percentile. Geary (2003) also argued that there are no universally agreed-upon criteria for the diagnosis of math difficulties.

4 The decision to sample one twentieth of these children was made so that we would have at least as many children in this group as in our second largest group (the group with poor reading and mathematics performance).
